# Acute kidney injury associated to sulfamethoxazole urine crystal: The importance of clinical suspicion

**DOI:** 10.5414/CNCS110931

**Published:** 2022-09-23

**Authors:** Rodrigo A. Sepúlveda, Fiorella Anghileri, Juan Pablo Huidobro E., Rodrigo Julio, Eduardo Ávila, Cristián Figueroa

**Affiliations:** 1 Nephrology Department, School of Medicine, Pontificia Universidad Católica de Chile, and; 2 Clinical Laboratories Department, Hospital Clínico Red de Salud UC-Christus, Santiago, Chile

**Keywords:** sulfamethoxazole, urine crystals, acute kidney injury, infrared spectroscopy

## Abstract

Management of acute kidney injury (AKI) associated with drug-induced crystal nephropathy can be difficult, and timely diagnosis is critical to resolve this condition. We present the case of a 55-year-old woman with history of systemic lupus erythematosus (SLE), who, after treatment with trimethoprim/sulfamethoxazole (TMP/SMX) for suspected *Pneumocystis jirovecii* pneumonia, developed severe AKI. Automated urinary sediment initially reported hematuria, leukocyturia and “uric acid crystals”. She did not have allergic symptoms, clinical manifestations of active SLE nor hyperuricemia. AKI persisted despite volume expansion with crystalloids. Due to SMX exposure, it was suspected that “uric acid crystals” could be in reality “SMX crystals”, and were a possible cause of crystal nephropathy. TMP/SMX was withheld and urinary alkalization was performed, with subsequent resolution of AKI. SMX urine crystals were posteriorly confirmed by Fourier transform infrared spectroscopy.

## Introduction 

Causes of acute kidney injury (AKI) are multiple, and prompt etiological diagnosis can be critical to establish appropriate treatment and prevent morbidity [1]. 

Identification of drug exposure is essential during evaluation of AKI, given the known renal susceptibility to multiple nephrotoxic agents. The mechanisms of kidney damage secondary to drugs are diverse [2]. Crystalline nephropathy secondary to medication exposure is not always explored due to its low frequency and complexities in diagnosis, but it is usually easily reversible [3]. Therefore, clinical suspicion and proper diagnostic protocols are crucial for the initiation of a timely treatment [4]. 

AKI guidelines recommend the use of urinalysis as part of the initial diagnostic approach. Automated urine analyzers are frequently used, with a performance comparable to manual analysis [5]. However, their diagnostic performance is unlikely to be adequate for detecting drug crystals compared to manual evaluation performed by a skilled operator [6, 7]. 

Due to the large list of drugs that can crystallize and precipitate in the nephron, and the limitations of automated urine tests, infrared spectroscopy remains the gold standard for the identification of urinary crystals [4, 8]. Fourier transform infrared spectroscopy (FT-IR) by attenuated total reflectance creates a unique “fingerprint” of each analyte related to its molecular structure [9]. It is easy to use and fast, but is expensive and requires sample preparation. Then, it is infrequently requested in a patient with AKI in the absence of clinical suspicion or urinary sediment findings of crystalluria. 

We report the case of a patient with AKI associated with sulfamethoxazole (SMX) urine crystals. After ruling out multiple causes of AKI, a crystal nephropathy due to SMX was presumed. Treatment directed against SMX crystalluria was performed, achieving complete renal recovery. 

## Case description 

**Figure 1.**
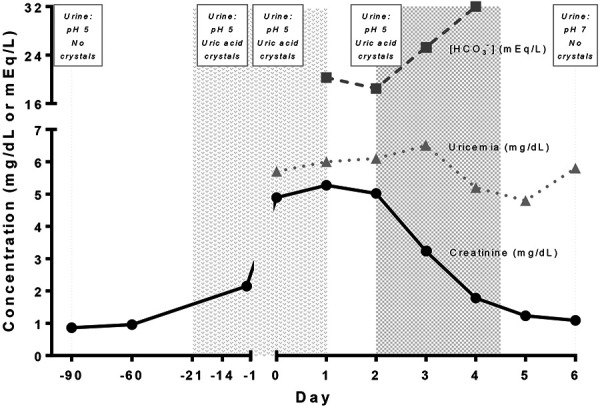
Patient development. Day –21: ambulatory evaluation when trimethoprim/sulfamethoxazole (TMP/SMX) was indicated for suspected pneumocystis. Day –14: emergency room consultation. Day –1: –ambulatory evaluation. Day 0: hospital admission. First shaded region: time exposed to SMX. Second shaded region: urinary alkalization was performed. All urinalysis was automated (both chemistry and microscopy). On day 2, urinalysis microscopy included a skilled medical technologist evaluation (from this sample, SMX crystals was obtained by transform infrared spectroscopy).

We report the case of a 55-year-old woman with past medical history of systemic lupus erythematosus (SLE) with exclusively articular involvement treated with prednisone and hydroxychloroquine. Two months before admission, she was hospitalized for COVID-19 pneumonia that responded to steroids and oxygen support, being discharged with 30 mg per day of prednisone and normal kidney function (Figure 1). The following month, in ambulatory medical control, she developed new respiratory symptoms and greater radiographic pulmonary involvement. A respiratory panel study (NxTAG Respiratory Pathogen Panel), *Pneumocystis jirovecii* in oral wash sample (LightCycler 480 Real-Time PCR System), and β-D-glucan were performed. Trimethoprim/sulfamethoxazole (TMP/SMX) 320/1,600 mg every 8 hours was initiated due to suspected pneumocystis pneumonia. One week later, the patient developed dysuria and decided to go to the emergency room. At admission, she had no fever or supplementary oxygen requirements. Computed tomography (CT) pulmonary angiogram and abdomen and pelvis CT scan showed parenchymal ground-glass opacities, signs of pulmonary organization, and early bilateral nephritis. Recent negative microbiological study was available. Automated urinalysis showed the following: pH 5.0, specific gravity 1.037, blood ++, leucocyte esterase +, glucose, protein, and nitrites negative. Automated urinary microscopy showed 15 red blood cells (RBC)/high power field (HPF), 11 white blood cells (WBC)/HPF, bacteria ++, and uric acid crystals ++. A diagnosis of urinary tract infection (UTI) and sequel of COVID-19 pneumonia were made. The patient was discharged maintaining prednisone and TMP/SMX, and cefadroxil was added to the treatment. Urine culture was positive for *Klebsiella pneumoniae*. Days later, in ambulatory control, new exams were evaluated highlighting creatinine of 2.15 mg/dL. The patient was then admitted to the hospital with diagnosis of AKI. 

She was admitted stable, without relevant findings at physical examination. Antimicrobials were discontinued and high-dose corticosteroids were maintained. Laboratory at admission was relevant for creatinine 4.89 mg/dL, blood urea nitrogen 32 mg/dL, Na^+^ 133 mEq/L, K^+^ 4.2 mEq/L, pH 7.41, HCO_3_^-^ 20.3 mEq/L, hemoglobin 9.7 g/dL, leukocytes 5,300/µL (0.2% eosinophils), uric acid 6 mg/dL, calcium 8.5 mg/dL, phosphate 5 mg/dL, and albumin 3.6 g/dL. In urine: protein/creatinine ratio 169 mg/g and FE_Na_ 3.7%. Automated urinalysis showed: pH 5.0, specific gravity 1.009, blood +++, leucocyte esterase, glucose, protein, and nitrites negative. Automated urinary sediment showed 50 – 100 RBC/HPF (no dysmorphia or acanthocytes), and uric acid crystals +. Urine culture was negative. Rheumatological laboratory showed normal C3 and C4, and negative anti-DNA. Non-contrast CT abdomen did not show ectasia. The next day, creatinine was 5.27 mg/dL associated with oliguria, despite administration of intravenous fluid. Physical examination did not show elements suggestive of active SLE or allergic interstitial nephritis. 

**Figure 2.**
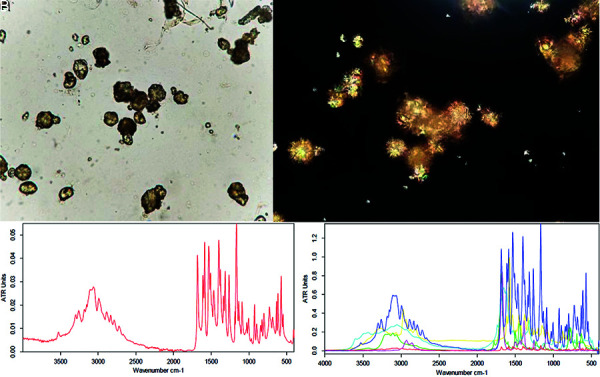
Sulfamethoxazole crystals. A: Bright field (× 40). B: Polarized light (× 40). C: Transform infrared spectroscopy (FT-IR) of the patient’s urinary crystals. D: Library spectra most similar to patient’s FT-IR: chloroquine (yellow, concordance 19.5%); sodium urate + whewellite + amorph. phosphate (light blue, concordance 20.2%); 4-phenylurazole (green, concordance 24.8%); N,N-dimethylformamide (pink, concordance 26.8%); N-acetylsulfamethoxazole (blue, concordance 96.4%).

Given the persistence of uric acid crystalluria, a manual review of the urinary sediment by an experienced observer was requested to evaluate the possibility of SMX crystals. The crystals had an irregular spheric shape and radial striations, yellow to brown color, and strong positive birefringence (Figure 2). With clinical suspicion of AKI associated to SMX crystals, urine alkalization with 1/6 molar sodium bicarbonate was performed. The patient responded favorably with complete resolution of AKI. 

Posterior analysis of the urine sediment with FT-IR confirmed the presence of SMX crystals (Figure 2). 

## Discussion 

Medications containing sulfonamides have been associated with the development of crystalluria, crystal nephropathy, and AKI. SMX crystalluria has been described in 0.4 – 49% of patients treated with TMP/SMX, with AKI in 11.2% of cases [10]. AKI generally develops within 7 days of treatment and should be considered in any patient exposed to TMP/SMX. A direct mechanism of renal damage by SMX crystals has not yet been established, but may be due to intratubular crystal deposition or allergic interstitial nephritis [11]. The risk of AKI is higher when sulfonamides are used in high doses, in situations of volume depletion, hypoalbuminemia, and in the presence of acidic urine, considering the acidic nature of SMX (pKa 6) and insolubility at pH < 5.5 [12, 13]. Our patient did not present hypoalbuminemia, but the recent use of iodinated contrast and UTI could have induced crystalluria. 

Routine search for crystalluria is proposed as part of the management in patients at risk of nephrotoxicity due to sulfonamides. SMX crystalluria can be prevented by avoiding high SMX doses, hypovolemia, and low urinary pH. Probably, intravenous fluid and urinary alkalization could be used to prevent and treat SMX crystalluria. When AKI associated with SMX crystalluria is suspected, SMX should be discontinued. 

To the best of our knowledge there are few reports of SMX crystalluria and AKI in the literature. Other reports have also described that SMX can simulate uric acid crystalluria in the urinary sediment [14]. This situation could influence a lower diagnosis of cases. SMX crystals are generally described as pleomorphic: appearance like “shocks or sheaves of wheat”, spheres, rosettes, fans and/or hexagons, among other forms, some of them very similar to those observed in this case report. Further, crystals can be yellow or brown, and by polarized light, are strongly birefringent [11, 13, 14, 15, 16]. Considering the variability of presentation of SMX crystals, and the possibility of misdiagnosis of uric acid crystals, the identification of SMX exposure is crucial for the correct diagnosis. 

The role of kidney biopsy for the diagnosis of SMX crystalluria is controversial since most of the histological findings are nonspecific (mild mononuclear infiltrate and tubulointerstitial fibrosis). SMX crystalluria have not been described in kidney tissue at pathology [11]. In the patient, FT-IR analysis of the pellet obtained from the centrifuged urine sample allowed to identify SMX crystals. The spectral curve was compatible with N-acetyl SMX, the main urinary metabolite of SMX [14]. 

AKI in the presented case could have had multiple causes. Renal hypoperfusion could be ruled out, since there was no history of fluid loss, no response to intravenous fluid, and no compatible FE_Na_. There was also no sepsis-induced AKI to explain the alterations. AKI progressed 2 weeks after the use of iodinated contrast, so this cause is unlikely. Intrarenal causes seemed likely: tubular injury due to drugs, acute interstitial nephritis, or lupus nephritis, were considered [17]. However, the absence of suggestive clinical and laboratory findings, as well as the absence of a response to steroidal treatment made these causes less likely. UTI had been treated for 2 weeks, which does not contraindicate a kidney biopsy; however, it was deferred to avoid higher infectious risks in an immunosuppressed patient [18]. Finally, biopsy was discarded due to the recovery of kidney function. The use of co-trimoxazole in therapeutic doses decreases the tubular secretion of creatinine, producing an increase in plasma levels of up to 23% in healthy subjects and up to 35% in chronic kidney disease [19]. However, the patient’s AKI is not explainable by this mechanism alone. We believe that the excellent response to urinary alkalization and suspension of TMP/SMX, together with suggestive urinalysis findings, supported the clinical presumption of acute SMX crystal nephropathy. 

## Conclusion 

SMX crystalluria could simulate uric acid crystals and is associated with AKI, possibly due to crystal nephropathy. This AKI may be preventable, reversible, but difficult to diagnose. Timely diagnosis is critical, requires clinical suspicion, and is favored by urinalysis performed by a skilled operator. Confirmation can be made with the use of FT-IR. 

## Acknowledgment 

We would like to thank Patricio Silva, Ana M. Guzmán, and Sandra Solari for their contribution in the study of this case. 

## Funding 

None. 

## Conflict of interest 

All authors have nothing to declare.
